# Supplementary material for: Ultrafast neural sampling with spiking nanolasers
Source: Nat Commun. 2025 Dec 3;17:125. doi: 10.1038/s41467-025-66818-1 (PMC12775008; doi:10.1038/s41467-025-66818-1)
Supplement: Supplementary file 1 — Supplementary Information [file 41467_2025_66818_MOESM1_ESM.pdf]

Supplementary Information
Ultrafast neural sampling with spiking nanolasers

Ivan K. Boikov[1,*], Alfredo de Rossi[1] and Mihai A. Petrovici[2]
[1] Thales Research & Technology, Palaiseau Cedex, 91767, France
[2] Department of Physiology, University of Bern, Bern, 3012, Switzerland
[*]mail@ikboikov.net

Supplementary Figures

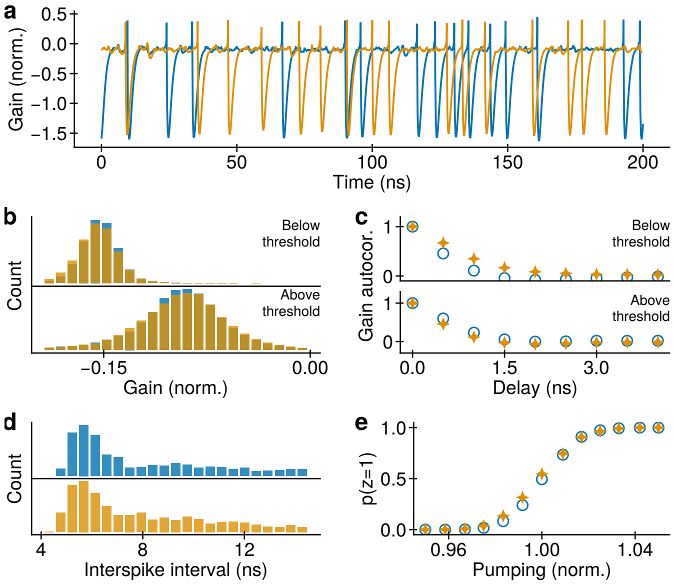

Supplementary Figure 1: Comparison of the simplified continuous model used throughout the article (blue) and the rigorous discrete model (orange). **(a)** Gain timetraces above the spiking threshold. **(b)** Distribution of gain outside the refractory period. Below and above the threshold correspond to normalized pumping of 0.96 and 1.0. **(c)** Same, autocorrelation of gain. **(d)** Interspike interval distribution for normalized pumping of 1.0. **(e)** Activation curves assuming identical τ used in the article.

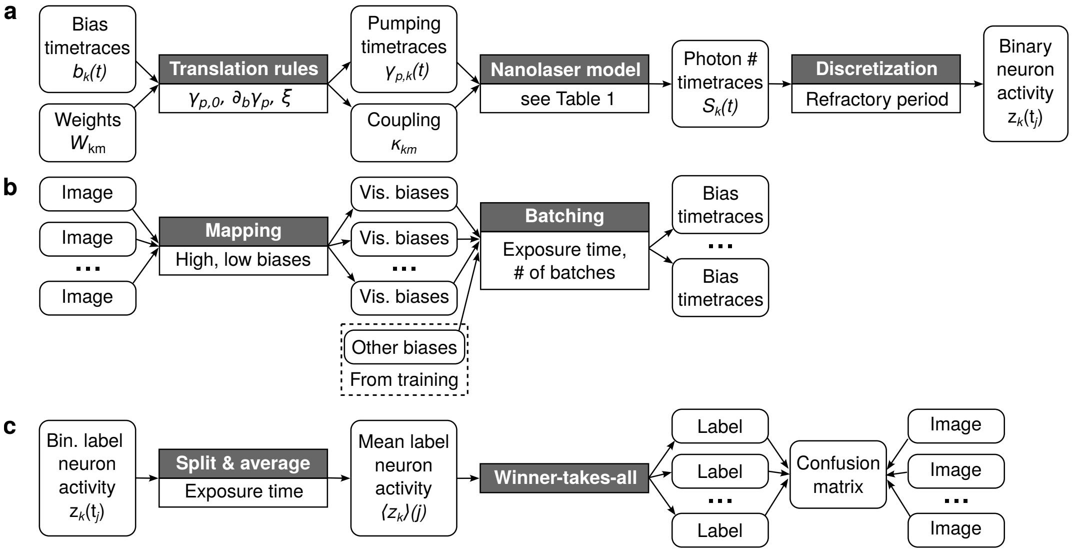

Supplementary Figure 2: Simulation code structure. **(a)** photonic spiking neuron (PSN) network simulation pipeline used for all tasks in the manuscript. **(b)** Preparation for MNIST classification with PSNs. Weights are used from the trained Boltzmann machine (BM). Batching was used to parallelize the simulation. **(c)** Processing of outputs from (b).

Supplementary Tables

Parameter	Value	Definition
γ	0.2 THz	photon damping rate
γ_{r}	$2.79 \times 10^{-6}\gamma$	transition rate
$\gamma_{\mathrm{r,a}}$	$5.31 \times 10^{-6}\gamma$	same, for the saturable absorber (SA)
γ_{t}	$1.28 \times 10^{-3}\gamma$	carrier damping rate
$\gamma_{\mathrm{t,a}}$	$1.01 \times 10^{-3}\gamma$	same, for the SA
χ_{g}	3	differential gain ratio
n_0	1.02×10^6	gain section dipole count
$n_{0,\mathrm{a}}$	8.20×10^5	same, in the SA

Supplementary Table 1: Parameters of the PSN model.

Parameter	Value
PSN operating point $\gamma_{\mathrm{p},0}$	1.0
Bias translation coeff. $\partial_u \gamma_{\mathrm{p}}$	0.0075
Weight translation coeff. ξ	$n_0/0.2$
Refractory period τ	11 ns
Exposure time (class.)	50τ
Exposure time (completion)	20τ
Exposure time (dream)	200τ
Low clamp bias	no pumping
High clamp bias	5.0

Supplementary Table 2: Simulation parameters.
